# Morphofunctional Outcomes of 3D Heads-Up Visualization Versus Standard Operating Microscopy in Idiopathic Epiretinal Membrane Surgery: A Prospective Randomized Study

**DOI:** 10.3390/jcm15187288

**Published:** 2026-09-19

**Authors:** Ludovico Iannetti, Lorenzo Sampalmieri, Alessio Speranzini, Arianna Barba, Magda Gharbiya, Marcella Nebbioso, Ludovico Alisi

**Affiliations:** 1 Policlinico Umberto I University Hospital, 00185 Rome, Italy; 2 Department of Sense Organs, Sapienza University of Rome, 00185 Rome, Italyarianna.barba@uniroma1.it (A.B.); ludovico.alisi@uniroma1.it (L.A.)

**Keywords:** epiretinal membrane, pars plana vitrectomy, 3D heads-up surgery, NGENUITY, standard operating microscope, multifocal electroretinography, OCT-based macular complications, endoillumination, vitreoretinal surgery

## Abstract

**Background/Objectives**: Three-dimensional (3D) heads-up visualization systems are increasingly used in vitreoretinal surgery and may offer advantages in intraoperative visualization, ergonomics, surgical education, and reduced light exposure. This study aimed to compare morphofunctional outcomes, intraoperative parameters, postoperative complications, and surgical team satisfaction between 3D heads-up visualization and standard operating microscopy in idiopathic epiretinal membrane (ERM) surgery. **Methods**: In this prospective randomized study, 25 eyes of 25 patients with idiopathic ERM were assigned to surgery using either a 3D heads-up visualization system (3D group, *n* = 13) or a standard operating microscope (SOM group, *n* = 12). BCVA, CMT, mfERG parameters, intraoperative variables, postoperative OCT-based macular complications, and satisfaction scores were evaluated. No formal sample-size calculation was performed; therefore, all analyses should be interpreted as exploratory and hypothesis-generating. **Results**: Postoperative BCVA and CMT were not significantly different between the two groups at both 1 and 3 months, suggesting that no major between-group difference was detected in final short-term visual and anatomical outcomes compared with standard operating microscopy in macular surgery. However, the 3D group had significantly worse preoperative BCVA, which may have inflated unadjusted change-score estimates. Therefore, the greater ΔBCVA observed in the 3D group should not be interpreted as evidence of superior efficacy. **Conclusions**: In idiopathic ERM surgery, 3D heads-up visualization showed no statistically significant difference in final anatomical and visual outcomes compared with standard operating microscopy, while allowing significantly lower endoillumination settings during peeling. The findings support its feasibility as an alternative visualization platform; however, they do not establish superiority, equivalence, or safety because of the exploratory design, small sample size, short follow-up, and baseline imbalances.

## 1. Introduction

Idiopathic epiretinal membrane (ERM) is a common vitreoretinal interface disorder characterized by fibrocellular proliferation on the inner retinal surface, which may lead to tangential macular traction, retinal distortion, decreased visual acuity, metamorphopsia, and impaired visual quality [1,2]. Pars plana vitrectomy with ERM and internal limiting membrane (ILM) peeling is the standard surgical approach in symptomatic cases, with the aim of relieving traction and improving retinal anatomy and function [2,3,4,5].

Because secondary ERMs may arise in association with retinal vascular disease, uveitis, retinal tears, trauma, retinal detachment, including complex rhegmatogenous retinal detachment with giant retinal tear, or previous vitreoretinal surgery, only idiopathic ERMs were included to reduce etiological heterogeneity [1,6].

In recent years, three-dimensional (3D) heads-up visualization systems have been increasingly adopted in vitreoretinal surgery [7,8,9,10,11,12]. The NGENUITY 3D Visualization System allows surgeons to operate while viewing a high-definition 3D monitor rather than using the oculars of a conventional operating microscope. This approach may provide several advantages, including improved ergonomics, shared visualization of the surgical field, enhanced teaching opportunities, and optimized image processing [9,11,12,13,14].

Previous studies comparing 3D heads-up visualization with standard operating microscopy in vitreoretinal surgery have reported comparable anatomical and functional outcomes, with potential advantages in surgeon comfort, depth perception, educational value, and reduced intraoperative illumination requirements [9,10,11,12,13,14,15,16,17,18,19]. Lower endoillumination may be particularly relevant during macular surgery, where prolonged light exposure to the posterior pole may theoretically contribute to retinal phototoxicity [8,20,21].

Most available studies have focused on visual acuity, anatomical outcomes, intraoperative feasibility, surgical time, and subjective satisfaction. However, limited evidence is available regarding electrophysiological retinal function after ERM surgery performed with 3D heads-up visualization. Multifocal electroretinography (mfERG) provides topographic information on macular function and may detect functional changes that are not fully captured by best-corrected visual acuity (BCVA) or optical coherence tomography (OCT) parameters alone [22,23,24,25,26].

The aim of this prospective randomized study was to compare the exploratory morphofunctional outcomes and feasibility of 3D heads-up visualization and standard operating microscopy in idiopathic ERM surgery. Specifically, we evaluated postoperative BCVA, CMT, mfERG changes, intraoperative parameters, postoperative OCT-based macular complications, and surgical team satisfaction.

## 2. Materials and Methods

### 2.1. Study Design and Participants

This prospective randomized study was conducted at Sapienza University of Rome, Policlinico Umberto I, between March 2022 and December 2025. The study adhered to the tenets of the Declaration of Helsinki. All participants provided informed consent before inclusion. The study was approved by the institutional ethics committee before recruitment. The incremental contribution of the present study lies in its prospective randomized design, exclusive focus on idiopathic ERM surgery, masked postoperative assessment, inclusion of mfERG as an objective functional outcome, and the absence of patient-level, operative, questionnaire, or electrophysiological data overlap with the previous related publication.

Eligible patients were randomly assigned before surgery in a 1:1 ratio to either the three-dimensional (3D) visualization group or the conventional operating microscope group. The allocation sequence was computer-generated using the random number function in Microsoft Excel. No block or stratified randomization was used. Patient enrollment was performed by study investigators, whereas the allocation sequence was maintained by a designated study member who was independent of patient enrollment and postoperative outcome assessment. The assigned surgical modality was communicated to the surgeon before surgery. All postoperative clinical evaluations were performed by investigators blinded to treatment allocation. OCT images were assessed by masked graders, and mfERG examinations and analyses were performed by personnel blinded to treatment allocation.

Inclusion criteria were diagnosis of stage II-IV idiopathic ERM according to the Govetto classification, documented reduction in visual acuity with or without metamorphopsia, absence of other retinal diseases potentially affecting visual function, axial length < 30 mm, age ≥ 40 years, and ability to comply with postoperative follow-up visits and study procedures.

Exclusion criteria were coexisting glaucoma, optic nerve disease, congenital ocular disorders, corneal opacities affecting visual acuity assessment, previous pars plana vitrectomy, secondary ERM related to retinal vascular occlusion, uveitis, diabetic retinopathy, or other retinal disease, axial length ≥ 30 mm, age < 40 years, severe systemic disease, and inability to complete follow-up.

### 2.2. Ophthalmic Assessment

All patients underwent a comprehensive ophthalmic evaluation before surgery, including best-corrected visual acuity (BCVA), slit-lamp biomicroscopy, intraocular pressure measurement using Goldmann applanation tonometry (Haag-Streit Group Gartenstadtstrasse 10, 3098 Koniz, Schweiz Switzerland), and indirect ophthalmoscopy. BCVA was measured using decimal charts and converted to logarithm of the minimum angle of resolution (logMAR) values for statistical analysis.

Spectral-domain optical coherence tomography (SD-OCT) scans were obtained using the Spectralis OCT system (Heidelberg Engineering, Heidelberg, Germany) according to a standardized acquisition protocol. Central macular thickness (CMT) was recorded preoperatively and during follow-up. All eyes underwent preoperative macular SD-OCT to assess ERM morphology and exclude pre-existing macular edema or intraretinal cystic changes. Thus, OCT-based macular complications recorded during follow-up represented new postoperative findings.

Postoperative examinations were performed at 1 day, 1 week, 1 month, and 3 months after surgery. The postoperative outcomes collected for the study included BCVA at 1 and 3 months, CMT at 1 and 3 months, mfERG parameters at 3 months, and postoperative complications.

### 2.3. Multifocal Electroretinography

All patients underwent multifocal electroretinography (mfERG) after instillation of 1% tropicamide in the study eye, followed by approximately 30 min of light adaptation. Recordings were obtained using a corneal contact electrode (ERG-Jet) after topical anesthesia with 0.5% benoxinate. The reference electrode was positioned at the ipsilateral outer canthus, and the ground electrode was placed on the earlobe using conductive gel. The fellow eye was occluded during testing.

Patients were positioned at a distance of 33 cm from the display, with near correction when required. The stimulus consisted of a pseudorandom sequence of 61 hexagonal elements alternating between light and dark, with a 50% probability of change at each frame. Signals were amplified and recorded digitally using the Vision Monitor MonPack 120 system (Metrovision, Perenchies, France), in accordance with the 2021 update of the International Society for Clinical Electrophysiology of Vision (ISCEV) standard for clinical mfERG [22].

The first-order kernel mfERG waveform was analyzed, including the initial negative deflection (N1), the positive peak (P1), and the second negative deflection (N2). The analysis focused on the P1 component. P1 amplitude and implicit time were evaluated in four eccentricity regions: the central 2°, 2–5°, 5–10°, and 10–15°. Only reliable mfERG recordings with adequate fixation and signal quality were included in the electrophysiological analysis.

### 2.4. Surgical Procedure

All surgeries were performed by two experienced vitreoretinal surgeons under peribulbar anesthesia using 5 mL mepivacaine 20 mg/mL and 5 mL ropivacaine 10 mg/mL. Both surgeons had more than 15 years of vitreoretinal surgical experience and had used the 3D visualization platform for approximately 4 years before the study. Both surgeons performed procedures using both visualization platforms. Surgeon 1 performed 14 procedures (5 SOM and 9 3D), whereas Surgeon 2 performed 11 procedures (7 SOM and 4 3D). Residents were present for educational purposes but did not perform surgical steps.

In the SOM group, surgery was performed using the LEICA F40 operating microscope (Leica Microsystems GmbH, Ernst-Leitz-Strasse 17–37, 35578 Wetzlar, Germany). In the 3D group, the microscope oculars were replaced with the NGENUITY 3D Visualization System (Alcon Laboratories, Fort Worth, TX, USA), which includes high dynamic range image acquisition, a 4K OLED 3D display, a high-speed image processor, and polarized 3D glasses.

All procedures were performed using the CONSTELLATION vitrectomy system (Alcon Laboratories, Fort Worth, TX, USA) with 25-gauge instruments. Triamcinolone acetonide (TAIOFTAL, Fidia Farmaceutici S.p.A., Abano Terme (PD), Italy) was used intraoperatively to visualize the vitreous. ERM and internal limiting membrane (ILM) staining was performed using a combination of Brilliant Blue G and Trypan Blue with 4% polyethylene glycol (TWIN Blue, Alchimia, Padova, Italy). ERM and ILM peeling were performed using standard microsurgical forceps.

Patients who were phakic at baseline underwent combined phacoemulsification and pars plana vitrectomy, whereas pseudophakic patients underwent vitrectomy alone. In combined cases, standard phacoemulsification was performed using the CENTURION system (Alcon Laboratories, Fort Worth, TX, USA), followed by implantation of a monofocal intraocular lens in the capsular bag. All patients received the same postoperative anti-inflammatory regimen, consisting of topical betamethasone/chloramphenicol eye drops tapered weekly from six daily administrations and topical bromfenac twice daily for 3 months.

### 2.5. Outcomes

The primary outcomes were changes in BCVA and mfERG parameters at 3 months after surgery. Secondary outcomes included changes in CMT, intraoperative parameters, postoperative OCT-based complications, and surgical team satisfaction scores.

The intraoperative variables recorded were total surgical time, total ERM peeling time, number of ERM flap initiations, dye exposure time, total ILM peeling time, number of ILM flap initiations, endoillumination levels during vitrectomy and membrane peeling, pupil diameter, and intraoperative complications. Total surgical time was defined as the interval from trocar insertion to closure of the sclerotomies; phacoemulsification time was therefore not included in this measure.

Postoperative complications were assessed clinically and by SD-OCT when appropriate. Complications included newly detected postoperative OCT-based macular complications, mainly characterized by macular edema and/or intraretinal or subretinal fluid on SD-OCT, retinal detachment, ocular hypertension, intraocular hemorrhage, and other postoperative adverse events.

### 2.6. Satisfaction Questionnaire

At the end of each procedure, the surgeon, resident, and scrub nurse completed a structured, non-validated satisfaction questionnaire evaluating the visualization technique used during surgery. The questionnaire assessed intraoperative visualization, ergonomics, surgical workflow, autonomy, teaching/learning opportunities, and team engagement using a 1–5 score, with higher scores indicating greater satisfaction. Questionnaires were completed for every case by one surgeon, one resident, and one scrub nurse, with no missing questionnaires. The resident raters consisted of three ophthalmology residents (two PGY3 and one PGY4), and the scrub nurse raters consisted of three ophthalmic operating-room nurses, each with more than 5 years of ophthalmic operating-room experience. The questionnaire items and scoring system are provided in Supplementary Table S1. These questionnaire outcomes were considered exploratory because the same team members may have rated multiple procedures and ratings were not masked to visualization platform.

### 2.7. Statistical Analysis

Continuous variables were expressed as median and interquartile range (IQR), while categorical variables were reported as frequencies and percentages. Normality was assessed using the Shapiro–Wilk test. Between-group comparisons were performed using the Mann–Whitney U test for continuous variables and Fisher’s exact test for categorical variables when appropriate. A two-sided nominal *p*-value < 0.05 was reported for exploratory purposes. No formal sample-size calculation was performed. Given the exploratory design and the large number of outcomes, *p*-values were interpreted as nominal and hypothesis-generating; no formal multiplicity correction was applied in the primary analysis. To assess the influence of the baseline BCVA imbalance, exploratory ANCOVA models were fitted with postoperative BCVA at 1 month and 3 months as dependent variables, treatment group as the independent variable (3D coded as 1 and SOM as 0), and baseline BCVA as a covariate. Adjusted group effects are reported as beta coefficients with 95% confidence intervals. More complex multivariable models including combined phaco-vitrectomy or surgeon were not considered statistically reliable because of the small sample size.

## 3. Results

**Figure 1:**
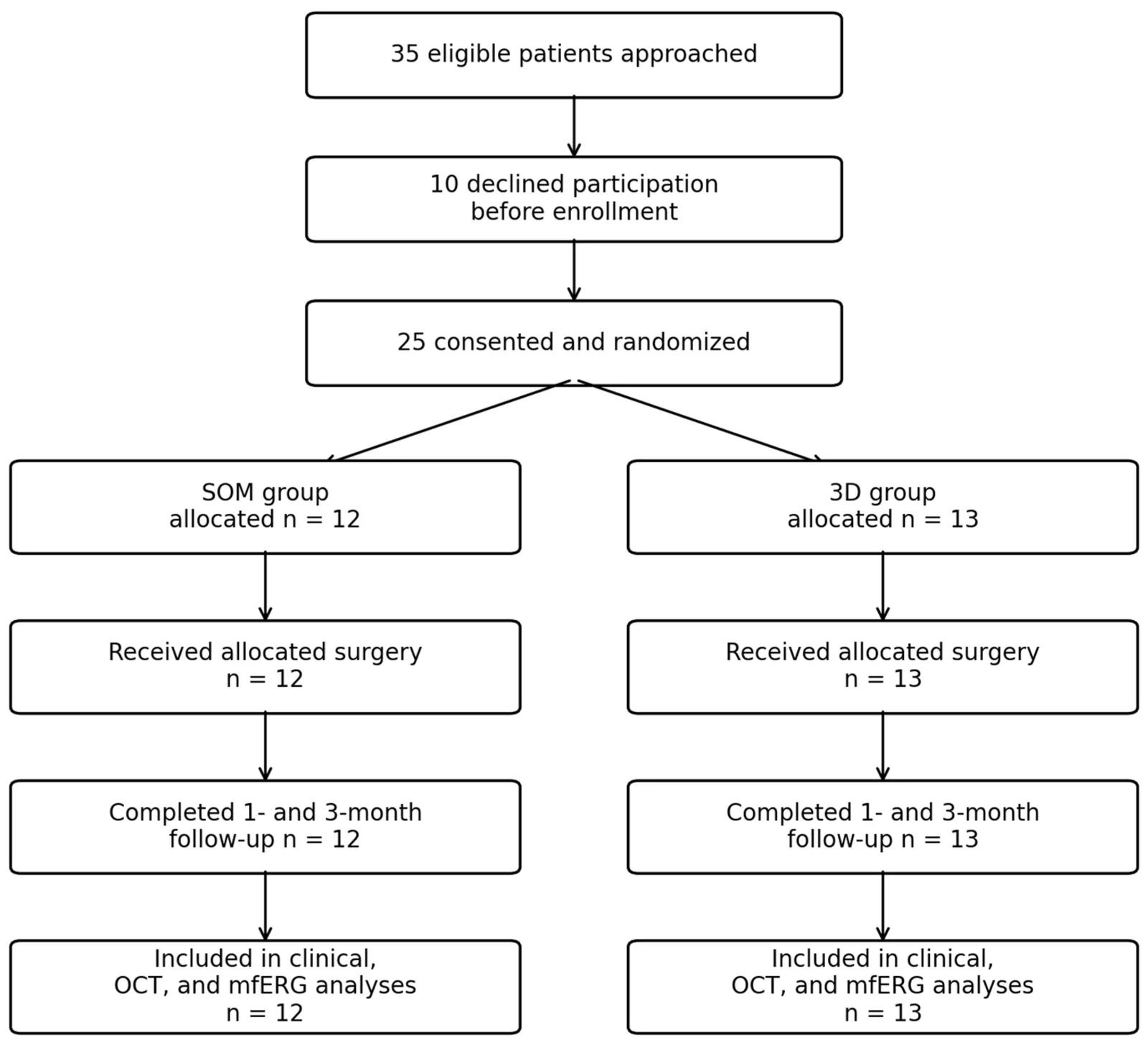
CONSORT-style participant flow diagram showing enrollment, randomization, follow-up, and analysis populations.

During the study period, 35 eligible patients were approached for participation; 10 declined participation and were not enrolled. The remaining 25 patients provided informed consent and were randomized: 12 were assigned to the SOM group and 13 to the 3D group. All randomized eyes received the assigned intervention, completed the scheduled 1-month and 3-month follow-up visits, and were included in the clinical, OCT, and mfERG analyses. The participant flow is summarized in Figure 1. Baseline clinical characteristics are summarized in Table 1. The two groups were comparable in terms of age, ERM stage, and preoperative CMT. However, preoperative BCVA was significantly worse in the 3D group than in the SOM group (0.7 (0.2) vs. 0.4 (0.125) logMAR; *p* = 0.002).

Intraoperative parameters are reported in Table 2. No statistically significant differences were observed between the two groups in total surgical time, ERM peeling time, ILM peeling time, number of flap initiations, dye exposure time, pupil diameter, or endoillumination during vitrectomy. Conversely, the 3D group required significantly lower endoillumination levels during membrane peeling compared with the SOM group (15 (5)% vs. 25 (6.25)%; *p* < 0.001).

No intraoperative complications occurred in either group.

**Figure 2:**
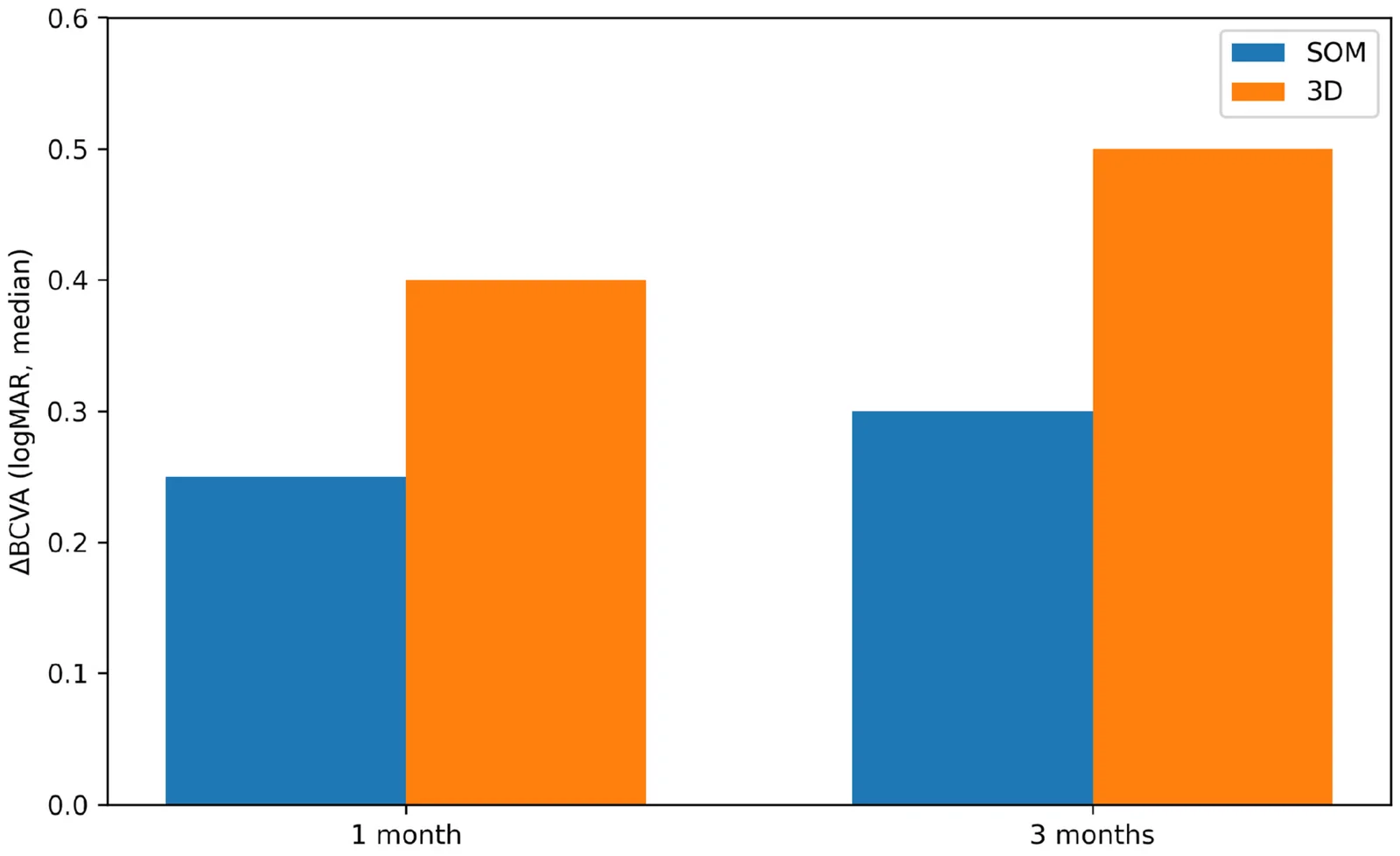
Visual recovery after surgery, expressed as change in logMAR best-corrected visual acuity (BCVA) from baseline, in the standard operating microscope (SOM) and 3D heads-up visualization groups at 1 and 3 months postoperatively. Bars represent median values; interquartile ranges are reported in Table 4. The figure is intended as a descriptive visual summary of median changes only; individual-level variability is reported in the corresponding table as interquartile range.

Postoperative functional and anatomical outcomes are shown in Table 3. At both 1 and 3 months after surgery, BCVA and CMT were not significantly different between groups, with no statistically significant between-group differences detected. Preoperative BCVA was significantly worse in the 3D group than in the SOM group (0.7 [0.2] vs. 0.4 [0.125] logMAR, *p* = 0.002). (Figure 2) Unadjusted ΔBCVA values were greater in the 3D group at 1 and 3 months (Table 4); however, these change-score findings were interpreted cautiously because baseline BCVA imbalance may influence the magnitude of apparent postoperative improvement. In exploratory ANCOVA models adjusted for baseline BCVA, the adjusted group effect for postoperative BCVA was −0.203 logMAR at 1 month (95% CI, −0.437 to 0.031; *p* = 0.086) and −0.123 logMAR at 3 months (95% CI, −0.251 to 0.005; *p* = 0.059), indicating that the apparent between-group advantage was attenuated after baseline adjustment.

**Figure 3:**
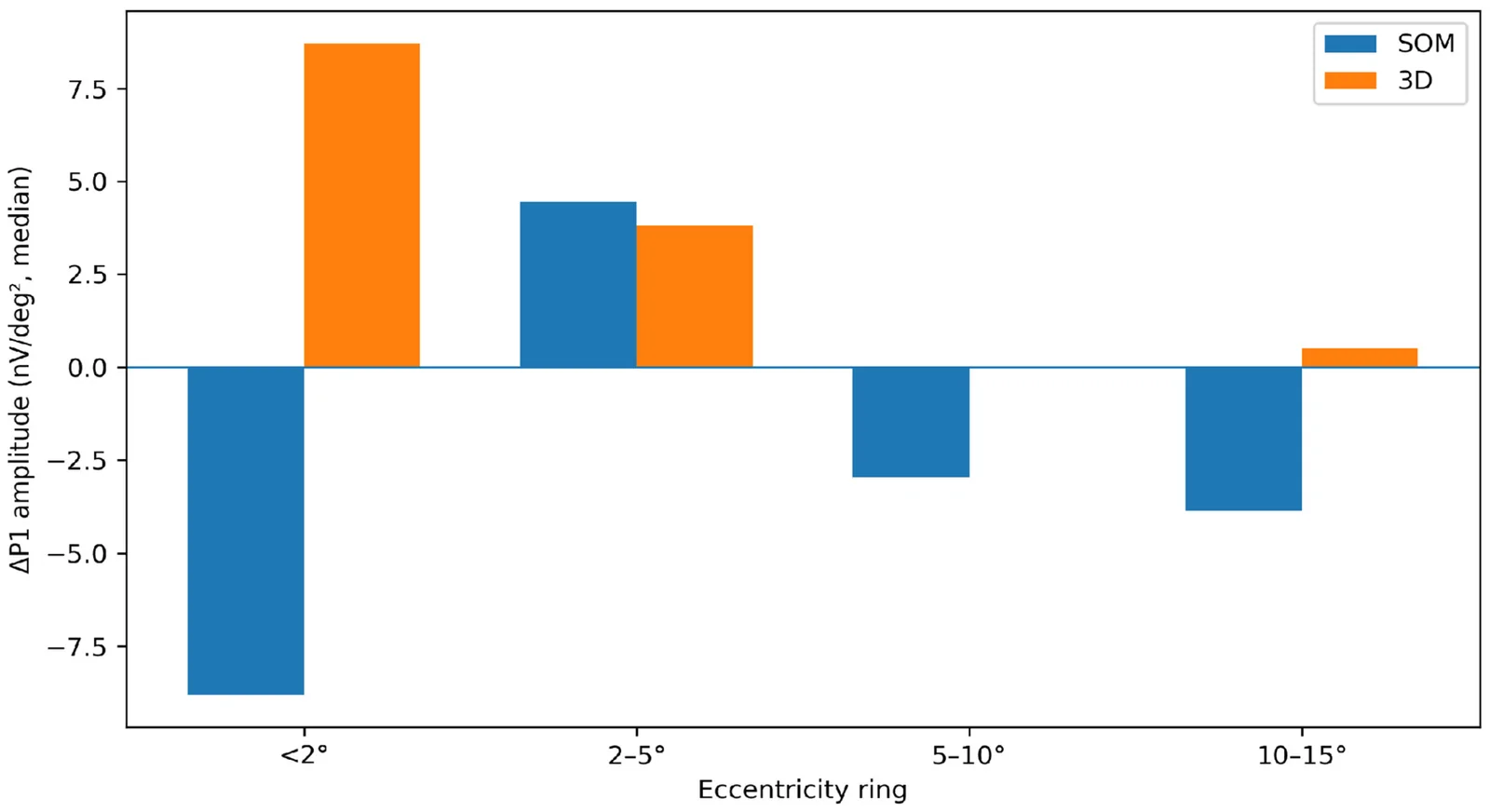
Changes in mfERG P1 amplitude after surgery across the four eccentricity rings. Bars represent median values; interquartile ranges are reported in Table 5. The figure is intended as a descriptive visual summary of median changes only; individual-level variability is reported in the corresponding table as interquartile range.

Changes in mfERG P1 amplitude and implicit time at 3 months after surgery are summarized in Table 5. mfERG analysis included 25 eyes (12 in the SOM group and 13 in the 3D group). (Figure 3) A nominal between-group difference was observed for ΔP1 amplitude in the 10–15° ring, favoring the 3D group (*p* = 0.007). No significant between-group differences were detected in the more central rings or in P1 implicit time. Given the number of mfERG comparisons, this isolated finding should be interpreted cautiously as exploratory and hypothesis-generating. Absolute baseline and 3-month postoperative mfERG values are reported in Supplementary Table S3.

Postoperative OCT-based macular complications are reported in Table 6 and summarized at case level in Supplementary Table S2. Complications were less frequent in the 3D group than in the SOM group (1/13, 7.7% vs. 5/12, 41.7%). These events were mainly characterized by newly detected postoperative macular edema and/or intraretinal or subretinal fluid on SD-OCT; no eye classified as having a postoperative OCT-based macular complication had pre-existing macular edema or intraretinal cystic changes on baseline SD-OCT. One SOM case initially labeled in the database as suspected CNV activation was adjudicated after chart and OCT review as postoperative OCT-based fluid/structural change without confirmed active neovascularization. This comparison was underpowered and potentially confounded by the imbalance in combined phaco-vitrectomy between groups.

Satisfaction questionnaire results are summarized in Table 7. Median scores were consistently higher for the 3D heads-up visualization system than for the standard operating microscope across most evaluated domains. Because the questionnaire was not validated, raters were not masked, and repeated ratings by the same team members may not have been statistically independent, these findings were considered descriptive and exploratory.

## 4. Discussion

This prospective randomized study compared morphofunctional outcomes, intraoperative parameters, postoperative OCT-based macular complications, and surgical team satisfaction between 3D heads-up visualization and standard operating microscopy in idiopathic ERM surgery. Because of the small sample size, baseline imbalance, and multiple exploratory outcomes, the findings should be interpreted as hypothesis-generating rather than confirmatory.

Postoperative BCVA and CMT did not differ significantly between groups at 1 and 3 months. This finding supports the feasibility of 3D heads-up visualization as an alternative platform but does not establish equivalence or superior efficacy. The 3D group showed greater BCVA improvement at 1 and 3 months; however, this finding should be interpreted cautiously because preoperative BCVA was significantly worse in this group. Patients with poorer baseline visual acuity may have greater potential for postoperative improvement, and this baseline imbalance may have contributed to the larger change observed in the 3D group. Therefore, no statistically significant between-group difference was detected in final short-term visual recovery, while the greater BCVA gain in the 3D group should be considered exploratory.

A key intraoperative finding was the significantly lower endoillumination setting used during ERM and ILM peeling in the 3D group. This is clinically relevant because macular surgery involves prolonged illumination near the posterior pole. However, retinal irradiance, illuminated retinal area, and cumulative light exposure were not directly measured; therefore, lower console illumination percentages should not be interpreted as direct proof of reduced phototoxicity. The potential phototoxicity advantage of 3D visualization remains theoretical in the present dataset and requires direct optical power/irradiance measurements in future studies.

Postoperative OCT-based macular complications were numerically less frequent in the 3D group than in the SOM group. Events were mainly characterized by postoperative macular edema and/or intraretinal or subretinal fluid on SD-OCT and were absent on preoperative OCT. Although this difference may be clinically relevant, it did not reach statistical significance and should not be interpreted as evidence that 3D visualization reduces postoperative complications.

Electrophysiological analysis showed a nominal between-group difference in P1 amplitude change in the 10–15° ring, favoring the 3D group. This isolated result should be interpreted cautiously because no significant between-group differences were observed in the more central rings or in P1 implicit time, and the analysis involved multiple correlated mfERG outcomes. Therefore, this finding should be considered exploratory and hypothesis-generating rather than evidence of superior macular functional recovery.

The satisfaction questionnaire showed consistently higher scores for the 3D heads-up visualization system across most evaluated domains. However, the questionnaire was non-validated, unmasked, and potentially affected by novelty/familiarity bias and pseudoreplication if the same team members rated multiple cases. Accordingly, these findings should be regarded as descriptive evidence of perceived usability and educational value, not as independent confirmatory outcomes.

This study has several limitations. First, the sample size was relatively small, limiting the statistical power, particularly for postoperative complications. No formal sample-size calculation was performed, and the study should therefore be considered an exploratory underpowered randomized study. Second, baseline BCVA was significantly worse in the 3D group, limiting interpretation of unadjusted visual recovery. Third, combined phaco-vitrectomy was more frequent in the SOM group and may have confounded postoperative visual and inflammatory outcomes. Fourth, mfERG analyses involved multiple correlated outcomes and should be interpreted cautiously because no formal multiplicity correction was applied. Fifth, questionnaire outcomes were subjective, non-validated, and potentially clustered by rater. Sixth, retinal irradiance, illuminated area, cumulative light exposure, and direct optical power were not measured, and no formal cost analysis was performed. Seventh, detailed mfERG acquisition parameters beyond those reported in the Methods were not available in the current dataset. Larger, adequately powered, preferably multicenter studies with standardized irradiance measurements, formal economic evaluation, longer follow-up, masked image grading, and prespecified statistical analysis are needed.

Overall, the present findings suggest that 3D heads-up visualization is a feasible alternative to standard operating microscopy in idiopathic epiretinal membrane surgery. They are also consistent with previous reports describing reduced endoillumination settings and favorable ergonomic and educational perception with 3D systems. However, the present study does not establish superiority, equivalence, or definitive safety.

## 5. Conclusions

In idiopathic epiretinal membrane surgery, the 3D heads-up visualization system showed no statistically significant difference in final short-term visual and anatomical outcomes compared with a standard operating microscope. The 3D system allowed lower endoillumination settings during peeling and was associated with higher exploratory satisfaction scores among the surgical team. Given the limited sample size and exploratory design, baseline BCVA imbalance, combined phaco-vitrectomy imbalance, and lack of direct retinal irradiance measurements, these findings should be interpreted as preliminary and hypothesis-generating. Although the present study provides encouraging preliminary evidence regarding the potential advantages of 3D visualization, further studies involving a larger cohort and, ideally, a prospective multicenter design would be useful to confirm these results.

## Figures and Tables

**Table 1:** Baseline clinical characteristics of patients undergoing idiopathic epiretinal membrane surgery using either the standard operating microscope or the 3D heads-up visualization system.

Variable	SOM Group (*n* = 12)	3D Group (*n* = 13)	*p*-Value
Age, years	74 (10.5)	76 (8)	0.643
Combined phaco-vitrectomy, *n* (%)	10 (83.3%)	7 (53.8%)	0.202
Pseudophakic vitrectomy, *n* (%)	2 (16.7%)	6 (46.2%)	-
Preoperative BCVA, logMAR	0.4 (0.125)	0.7 (0.2)	0.002
Preoperative CMT, µm	452 (64)	422 (102)	0.644
ERM stage II, *n* (%)	2 (16.7%)	4 (30.8%)	-
ERM stage III, *n* (%)	5 (41.7%)	6 (46.2%)	-
ERM stage IV, *n* (%)	5 (41.7%)	3 (23.1%)	-

Data are presented as median (interquartile range) unless otherwise specified. Categorical variables are presented as *n* (%). The *p*-value for lens/surgical status was calculated using Fisher’s exact test. SOM = standard operating microscope; 3D = three-dimensional heads-up visualization system; ERM = epiretinal membrane; BCVA = best-corrected visual acuity; CMT = central macular thickness.

**Table 2:** Intraoperative parameters in the standard operating microscope and 3D heads-up visualization groups.

Variable	SOM Group (*n* = 12)	3D Group (*n* = 13)	*p*-Value
Surgical time, min	23 (7)	22 (10)	0.891
Total ERM peeling time, min	3.65 (2.1)	3.5 (3)	0.848
ERM flap initiations, *n*	2.5 (1)	2 (1)	0.931
Dye exposure time, sec	30 (70)	90 (60)	0.510
Total ILM peeling time, min	3 (2.25)	3.5 (2)	0.721
ILM flap initiations, *n*	3 (2)	3 (1)	0.353
Endoillumination during vitrectomy, %	27.5 (10)	30 (5)	0.595
Endoillumination during peeling, %	25 (6.25)	15 (5)	<0.001
Pupil diameter, mm	6.5 (1.25)	6 (1)	0.819

Data are presented as median (interquartile range). SOM = standard operating microscope; 3D = three-dimensional heads-up visualization system; ERM = epiretinal membrane; ILM = internal limiting membrane.

**Table 3:** Postoperative functional and anatomical outcomes at 1 and 3 months after surgery.

Variable	SOM Group (*n* = 12)	3D Group (*n* = 13)	*p*-Value
BCVA at 1 month, logMAR	0.1 (0.2)	0.2 (0.3)	0.715
BCVA at 3 months, logMAR	0 (0.2)	0.1 (0.2)	0.596
CMT at 1 month, µm	433 (41.75)	408 (45)	0.242
CMT at 3 months, µm	419.5 (63.5)	386 (39)	0.128
ΔCMT 1 month, µm	27.5 (90.5)	40.0 (67.0)	0.849
ΔCMT 3 months, µm	42.5 (97.5)	38.0 (85.0)	0.446

Data are presented as median (interquartile range). ΔCMT was calculated as baseline CMT minus postoperative CMT; therefore, positive values indicate a reduction in central macular thickness. SOM = standard operating microscope; 3D = three-dimensional heads-up visualization system; BCVA = best-corrected visual acuity; CMT = central macular thickness.

**Table 4:** Visual recovery after surgery, expressed as change in logMAR BCVA from baseline.

Variable	SOM Group (*n* = 12)	3D Group (*n* = 13)	*p*-Value
ΔBCVA at 1 month, logMAR	0.25 (0.3)	0.4 (0.1)	0.012
ΔBCVA at 3 months, logMAR	0.3 (0.22)	0.5 (0.3)	0.002

Data are presented as median (interquartile range). ΔBCVA was calculated as baseline logMAR BCVA minus postoperative logMAR BCVA; therefore, positive values indicate visual improvement. SOM = standard operating microscope; 3D = three-dimensional heads-up visualization system; BCVA = best-corrected visual acuity.

**Table 5:** Changes in mfERG P1 amplitude and implicit time at 3 months after surgery.

Variable	SOM Group (*n* = 12)	3D Group (*n* = 13)	*p*-Value
ΔP1 amplitude < 2°, nV/deg^2^	−8.8 (23.5)	8.7 (26.3)	0.087
ΔP1 amplitude 2–5°, nV/deg^2^	4.45 (21.45)	3.8 (13.8)	0.892
ΔP1 amplitude 5–10°, nV/deg^2^	−2.95 (10.57)	−0.01 (6.8)	0.221
ΔP1 amplitude 10–15°, nV/deg^2^	−3.85 (1.74)	0.5 (5.8)	0.007 (nominal)
Delta P1 implicit time < 2°, ms	−0.75 (6.02)	0.90 (7.2)	0.253
Delta P1 implicit time 2–5°, ms	−0.55 (3.68)	0.10 (3.11)	0.978
Delta P1 implicit time 5–10°, ms	−0.55 (1.45)	−0.70 (1.8)	1.000
Delta P1 implicit time 10–15°, ms	−0.80 (1.79)	−0.90 (2.6)	0.870

Data are presented as median (interquartile range). SOM = standard operating microscope; 3D = three-dimensional heads-up visualization system; mfERG = multifocal electroretinography. Absolute baseline and 3-month postoperative mfERG values are provided in Supplementary Table S3.

**Table 6:** Postoperative OCT-based macular complications in the standard operating microscope and 3D heads-up visualization groups.

Variable	SOM Group (*n* = 12)	3D Group (*n* = 13)
No postoperative OCT-based macular complications, *n* (%)	7 (58.3%)	12 (92.3%)
Postoperative OCT-based macular complications, *n* (%)	5 (41.7%)	1 (7.7%)

Categorical variables are presented as *n* (%). Fisher’s exact test, *p* = 0.073. The exploratory risk difference for postoperative OCT-based macular complications (3D minus SOM) was −34.0 percentage points (Newcombe 95% CI, −61.1 to +0.02 percentage points). This estimate should be interpreted cautiously because of the small number of events and the imbalance in combined phaco-vitrectomy between groups. SOM = standard operating microscope; 3D = three-dimensional heads-up visualization system.

**Table 7:** Satisfaction questionnaire scores for the surgeon, resident, and scrub nurse.

Domain/Item	3D Median	3D IQR	SOM Median	SOM IQR
**Surgeon**				
Trocar insertion	5	1	3	0.5
Vitrectomy	4	1	2	1.25
ILM peeling	5	1	2	1.25
Insertion of tamponade	4	2	3	0.5
Closing	5	1	3	1
Overall ergonomics	5	1	2	1.25
Resident autonomy	5	1	2	1
Awareness of other team members	5	1	2	0.75
Pointing out landmarks	5	0	2	0.75
**Resident**				
Learning opportunity	5	1	3	0
Understanding anatomy	5	1	3	0.25
Identifying surgical targets	4	1	3	1.5
Asking questions	4	1	2	0.75
Overall ergonomics	5	0	2	0.5
Autonomy	5	1	2	0
**Scrub nurse**				
Case set-up	5	2	3	1
Tracking surgical progress	5	2	2	1
Anticipating instruments	5	1	2	0
Engagement	5	1	3	0.25

Scores ranged from 1 to 5, with higher scores indicating greater satisfaction. Data are presented as median and interquartile range. SOM = standard operating microscope; 3D = three-dimensional heads-up visualization system; ILM = internal limiting membrane.

## Data Availability

The de-identified datasets generated and/or analyzed during the current study are not publicly available due to privacy and ethical restrictions regarding patient confidentiality but are available from the corresponding author upon reasonable request.

## Supplementary Materials

The following supporting information can be downloaded at: https://www.mdpi.com/article/10.3390/jcm15187288/s1, Table S1. Satisfaction questionnaire items and scoring system; Table S2. Case-level description of postoperative OCT-based macular complications; Table S3. Absolute mfERG P1 response amplitude and implicit time values at baseline and 3 months after surgery.

## References

[1] Fung A.T., Galvin J., Tran T. (2021). Epiretinal membrane: A review. Clin. Exp. Ophthalmol..

[2] Iuliano L., Fogliato G., Gorgoni F., Corbelli E., Bandello F., Codenotti M. (2019). Idiopathic epiretinal membrane surgery: Safety, efficacy and patient-related outcomes. Clin. Ophthalmol..

[3] Govetto A., Lalane R.A., Sarraf D., Figueroa M.S., Hubschman J.P. (2017). Insights into epiretinal membranes: Presence of ectopic inner foveal layers and a new optical coherence tomography staging scheme. Am. J. Ophthalmol..

[4] Govetto A., Virgili G., Rodriguez F.J., Figueroa M.S., Sarraf D., Hubschman J.P. (2019). Functional and anatomical significance of the ectopic inner foveal layers in eyes with idiopathic epiretinal membranes: Surgical results at 12 months. Retina.

[5] Kim J.H., Kim Y.M., Chung E.J., Lee S.Y., Koh H.J. (2012). Structural and functional predictors of visual outcome of epiretinal membrane surgery. Am. J. Ophthalmol..

[6] Lee S., Kim J. (2024). Rhegmatogenous retinal detachment with giant retinal tear: Case series and literature review. J. Clin. Med..

[7] Eckardt C., Paulo E.B. (2016). Heads-up surgery for vitreoretinal procedures: An experimental and clinical study. Retina.

[8] Adam M.K., Thornton S., Regillo C.D., Park C., Ho A.C., Hsu J. (2017). Minimal endoillumination levels and display luminous emittance during three-dimensional heads-up vitreoretinal surgery. Retina.

[9] Talcott K.E., Adam M.K., Sioufi K., Aderman C.M., Ali F.S., Mellen P.L., Garg S.J., Hsu J., Ho A.C. (2019). Comparison of a three-dimensional heads-up display surgical platform with a standard operating microscope for macular surgery. Ophthalmol. Retina.

[10] Zhang T., Tang W., Xu G. (2019). Comparative analysis of three-dimensional heads-up vitrectomy and traditional microscopic vitrectomy for vitreoretinal diseases. Curr. Eye Res..

[11] Kantor P., Matonti F., Varenne F., Sentis V., Pagot-Mathis V., Fournié P., Soler V. (2021). Use of the heads-up NGENUITY 3D Visualization System for vitreoretinal surgery: A retrospective evaluation of outcomes in a French tertiary center. Sci. Rep..

[12] Razavi P., Cakir B., Baldwin G., D’Amico D.J., Miller J.B. (2023). Heads-up three-dimensional viewing systems in vitreoretinal surgery: An updated perspective. Clin. Ophthalmol..

[13] Rizzo S., Abbruzzese G., Savastano A., Giansanti F., Caporossi T., Barca F., Faraldi F., Virgili G. (2018). 3D surgical viewing system in ophthalmology: Perceptions of the surgical team. Retina.

[14] Romano M.R., Cennamo G., Comune C., Cennamo M., Ferrara M., Rombetto L., Cennamo G. (2018). Evaluation of 3D heads-up vitrectomy: Outcomes of psychometric skills testing and surgeon satisfaction. Eye.

[15] Kim D.J., Kim D.G., Park K.H. (2023). Three-dimensional heads-up vitrectomy versus conventional microscopic vitrectomy for patients with epiretinal membrane. Retina.

[16] Giansanti F., Nicolosi C., Bacherini D., Vicini G., Russo C., Scialdone A., Caporossi T., Savastano A. (2023). Three-dimensional visualization system for vitreoretinal surgery: Results from a monocentric experience and comparison with conventional surgery. Life.

[17] Shoshany T.N., Agranat J.S., Armstrong G., Miller J.B. (2020). The user experience on a 3-dimensional heads-up display for vitreoretinal surgery across all members of the health care team: A survey of medical students, residents, fellows, attending surgeons, nurses, and anesthesiologists. J. Vitreoretin. Dis..

[18] Zhao X., Zhao Q., Hu H., Guo J., Lv H., Chen Y. (2024). Comparison of three-dimensional heads-up system versus traditional microscopic system in medical education for vitreoretinal surgeries: A prospective study. BMC Med. Educ..

[19] Iannetti L., Baratta C., Romaniello A., Magnolo C., Ruggeri F., Blasi F.R., Carlesimo S.C., Gharbiya M., Scarinci F., Alisi L. (2026). Enhanced Surgical Efficiency with 3D Heads-Up Visualization in Vitreoretinal Surgery: A Retrospective Comparative Study. J. Clin. Med..

[20] Michels M., Lewis H., Abrams G.W., Han D.P., Mieler W.F., Neitz J. (1992). Macular phototoxicity caused by fiberoptic endoillumination during pars plana vitrectomy. Am. J. Ophthalmol..

[21] Fehler N., Lingenfelder C., Hessling M., Kupferschmid S. (2023). Retinal risk of endoillumination: A comparison of different ophthalmic illumination systems. J. Fr. Ophtalmol..

[22] Hoffmann M.B., Bach M., Kondo M., Li S., Walker S., Holopigian K., Viswanathan S., Robson A.G. (2021). ISCEV standard for clinical multifocal electroretinography (mfERG) (2021 update). Doc. Ophthalmol..

[23] Moschos M., Apostolopoulos M., Ladas J., Theodossiadis P., Malias J., Moschos M., Papaspirou A., Theodossiadis G. (2001). Assessment of macular function by multifocal electroretinogram before and after epimacular membrane surgery. Retina.

[24] Shimada Y., Sakurai S., Naito K., Sugino T., Yoshioka M., Matsumoto M., Kado M., Ito T. (2011). Multifocal electroretinogram and optical coherence tomography: Prediction of visual outcome after epiretinal membrane removal. Clin. Exp. Optom..

[25] Gao M., Wang Y., Liu W., Liu L., Yan W., Liu J., Liu K., Liu X., Hu Y. (2017). Assessment of macular function in patients with idiopathic epiretinal membrane by multifocal electroretinography: Correlation with visual acuity and optical coherence tomography. BMC Ophthalmol..

[26] Lim J.W., Cho J.H., Kim H.K. (2010). Assessment of macular function by multifocal electroretinography following epiretinal membrane surgery with internal limiting membrane peeling. Clin. Ophthalmol..

