# Supplementary material for: Morphofunctional Outcomes of 3D Heads-Up Visualization Versus Standard Operating Microscopy in Idiopathic Epiretinal Membrane Surgery: A Prospective Randomized Study
Source: J Clin Med. 2026 Sep 19;15(18):7288. doi: 10.3390/jcm15187288 (PMC13607525; doi:10.3390/jcm15187288)
Supplement: Supplementary file 1 [file jcm-15-07288-s001.zip › jcm-4448796-supplementary.pdf]

Supplementary Materials

Supplementary Table S1. Satisfaction questionnaire items and scoring system.

**Role/domain**	**Questionnaire item**	**Score**
Surgeon	Trocar insertion	1-5
Surgeon	Vitrectomy	1-5
Surgeon	ILM peeling	1-5
Surgeon	Insertion of tamponade	1-5
Surgeon	Closing	1-5
Surgeon	Overall ergonomics	1-5
Surgeon	Resident autonomy	1-5
Surgeon	Awareness of other team members	1-5
Surgeon	Pointing out landmarks	1-5
Resident	Learning opportunity	1-5
Resident	Understanding anatomy	1-5
Resident	Identifying surgical targets	1-5
Resident	Asking questions	1-5
Resident	Overall ergonomics	1-5
Resident	Autonomy	1-5
Scrub nurse	Case set-up	1-5
Scrub nurse	Tracking surgical progress	1-5
Scrub nurse	Anticipating instruments	1-5
Scrub nurse	Engagement	1-5

Scores ranged from 1 to 5, with higher scores indicating greater satisfaction. The questionnaire was structured for this study and was not formally validated.

Supplementary Table S2. Case-level description of postoperative OCT-based macular complications.

**Case ID**	**Group**	**Surgery type**	**Onset**	**OCT phenotype / final adjudication**	**Treatment**	**Resolution**
SOM-1	SOM	Vitrectomy alone (pseudophakic)	1 month; increased at 3 months	Postoperative OCT-based macular edema	Not recorded in study database	Not recorded in study database
SOM-2	SOM	Combined phaco-vitrectomy	3 months	Postoperative OCT-based macular edema	Not recorded in study database	Not recorded in study database
SOM-3	SOM	Combined phaco-vitrectomy	3 months	Initially labeled suspected CNV activation; final review did not confirm active neovascularization	Not recorded in study database	Not recorded in study database
SOM-4	SOM	Combined phaco-vitrectomy	1 month	Postoperative macular edema	Ozurdex implant	Not recorded in study database
SOM-5	SOM	Combined phaco-vitrectomy	3 months	Postoperative macular edema;	Not recorded in study database	Not recorded in study database

				postoperative ocular hypertension also recorded		
3D-1	3D	Combined phaco-vitrectomy	1 month	Mild postoperative macular edema	Topical anti-inflammatory therapy	Resolved

Supplementary Table S3. Absolute mfERG P1 response amplitude and implicit time values at baseline and 3 months after surgery.

Panel A. P1 response amplitude (nV/deg^2).

Eccentricity ring	Time point	SOM (n = 12)	3D (n = 13)	p-value
<2°	Baseline	45.6 (29.9)	52.9 (25.5)	0.064
<2°	3 months	46.1 (12.0)	55.9 (28.3)	0.744
2-5°	Baseline	46.9 (5.0)	48.7 (13.1)	0.496
2-5°	3 months	41.5 (12.9)	47.3 (10.6)	0.765
5-10°	Baseline	37.9 (14.2)	41.2 (10.4)	0.807
5-10°	3 months	46.7 (15.4)	42.4 (9.1)	0.183
10-15°	Baseline	30.7 (7.2)	34.0 (7.1)	0.605
10-15°	3 months	36.7 (12.9)	31.7 (5.1)	0.174

Panel B. P1 implicit time (ms).

Eccentricity ring	Time point	SOM (n = 12)	3D (n = 13)	p-value
<2°	Baseline	46.4 (4.6)	47.6 (3.9)	0.531
<2°	3 months	47.8 (1.4)	46.3 (3.6)	0.220
2-5°	Baseline	47.4 (6.0)	46.8 (3.0)	0.978
2-5°	3 months	47.5 (1.4)	47.1 (3.2)	0.913
5-10°	Baseline	45.6 (3.6)	45.8 (2.0)	0.765
5-10°	3 months	47.0 (2.1)	46.6 (2.6)	0.849
10-15°	Baseline	45.4 (2.8)	45.7 (1.6)	0.807
10-15°	3 months	46.8 (2.5)	46.3 (1.4)	0.806

Data are presented as median (interquartile range). Between-group p-values were calculated using the Mann-Whitney U test. SOM = standard operating microscope; 3D = three-dimensional heads-up visualization system; mfERG = multifocal electroretinography.
